# A Comparative Evaluation of Real-Time Guided Dynamic Navigation and Conventional Techniques for Post Space Preparation During Post Endodontic Management: An In Vitro Study

**DOI:** 10.7759/cureus.66900

**Published:** 2024-08-14

**Authors:** Sherifa Shervani, Sihivahanan Dhanasekaran, Vijay Venkatesh

**Affiliations:** 1 Department of Conservative Dentistry and Endodontics, Sri Ramaswamy Memorial (SRM) Kattankulathur Dental College and Hospital, Chennai, IND

**Keywords:** real-time guided endodontics, navident, post endodontic management, post space preparation, guided endodontics, dynamic navigation

## Abstract

Introduction: The three-dimensional (3D) dynamic navigation system (DNS; Navident, ClaroNav Technology, Toronto, ON) is a revolutionary technique in endodontics that offers superior precision and efficiency compared to existing techniques for post space preparation.

Aim: The aim is to evaluate and contrast the efficacy and efficiency of the DNS with conventional post space preparation techniques. This assessment considers several parameters, notably canal deviation (global coronal and apical deviation and angular deflection), duration of the procedure, and total volumetric loss of instrumented root canal and volumetric loss of instrumented root canal above 4 mm from the apex.

Materials and methods: Freshly extracted maxillary central incisors were chosen for this study. A total sample size of 60 (n) was included. The extracted teeth samples were divided into two groups: 3D DNS (group I; n = 30) and conventional techniques (group II; n = 30). The samples were taken, and 50% of the crown structure was reduced for post space preparation to ensure standardization between the two groups. The samples were root canal-treated and mounted in a 3D-printed maxillary cast. Preoperative micro-computed tomography (micro-CT) and cone-beam computed tomography (CBCT) were taken for both groups. For group I, post space preparation was conducted with the DNS, which provided comprehensive guidance. The procedure was stopped when post space preparation was 4 mm short of the apex, as indicated in the system display. For group II, post space preparation was done without the guidance of DNS. Time taken for the procedure was assessed using a timer; canal deviation was evaluated using CBCT analysis, and volumetric loss was estimated using micro-CT analysis.

Results: The dynamic navigation group achieves significantly more precise outcomes in post space preparation than the conventional technique. The DNS group has significantly lesser global coronal and apical deviation and angular deflection compared to the conventional group (p < 0.05). The DNS group has reduced the volumetric loss of instrumented root canals compared to the conventional group (p < 0.05). Furthermore, the DNS group requires significantly less time than the conventional method, with a mean difference of about 10.567 minutes (p < 0.05).

Conclusion: Implementing dynamic navigation improves precision in post space preparation, with a notable reduction in canal deviation and volumetric loss and a decrease in procedure time compared to the conventional method.

## Introduction

The prolonged existence of teeth that have undergone root canal therapy relies on maintaining the structural integrity of the tooth substance. Posts are primarily used to reinforce the retention or stability of dental restorations in the coronal portion of the tooth. They are often recommended for teeth with substantial loss of tooth structure in the coronal region. The result of post-retained restorations, to some degree, depends on ferrule design, overall remaining tooth structure, and kind of eventual replacement [1,2]. Several suggestions have been made on the characteristics of post space preparation, including techniques for eliminating obturation material and expanding the canal space. Various methods have been proposed to remove the current filling material in the root canal to create room for the post. These procedures include using chemical solvents, heated devices, and spinning equipment such as Gates-Glidden drills or Peeso reamers. While manual extraction of gutta-percha is often used and shown to be efficient, it may result in substantial harm to the root canal walls and the temporary formation of regions of intense stress in the dentin owing to contact between the instrument and the dentin walls. Utilizing more rigid or larger instruments enhances the level of contact with the surface, increases friction, and intensifies the concentration of stress. This may result in harm to the dentin or the formation of partial cracks, which may eventually develop into vertical root fractures (VRFs), thus affecting the tooth's long-term survival. Although VRFs are often seen in teeth with intraradicular posts, less attention seems to be given to the formation of dentin defects resulting from the influence of post space preparation. Studies have shown that augmenting the diameters and lengths of posts does not improve fracture resistance or the ability of the post to stay in place. Hence, it is advisable to ensure that the width of the post space is no more than one-third of the root diameter and that the remaining dentin has a minimum thickness of 1 mm. This must be considered during the post space preparation procedure [3]. The dynamic navigation system (DNS; Navident, ClaroNav Technology, Toronto, ON) is a novel 3-dimensional (3D) technology that has been investigated in the area of endodontics. It shows potential for improving the accuracy and safety of endodontic treatments while reducing risks. The 3D-DNS involves using a cone-beam computed tomography (CBCT) scan for preoperative virtual planning and computer-based monitoring throughout treatment. The DNS acts as a global navigation system for drills and handpieces, enabling fast and customizable navigation and visual guidance to direct instruments throughout the procedure [4]. Real-time guided endodontics significantly reduces tooth substance loss, making it a more dependable and efficient alternative to conventional procedures. Moreover, unlike traditional preparations, the operator's experience has no bearing on the effectiveness of the guided procedure [5]. By precisely determining the possible angle at which the reamers will remove the gutta-percha, the DNS increases the benefits of post space preparation. This approach enhances the removal of gutta-percha while minimizing tooth tissue damage and the risk of iatrogenic injury or perforation [6]. From a clinical perspective, assessing the effectiveness of post space preparation involves determining the duration of the procedure until no visible radiopaque remnants are seen on radiographs, particularly within a distance of 4-5 mm from the apex. In addition, the evaluation includes the analysis of canal deviation by measurement of global coronal and apical deviation and angular deflection during the preparation of post space by CBCT. The analysis further includes assessing volumetric loss in the canal space through micro-computed tomography (micro-CT). A survey of the existing literature reveals a lack of previous research that has compared the DNS with traditional ways in the context of post space preparation during post endodontic treatment. Consequently, the primary objective of this research is to assess and contrast the efficacy and efficiency of conventional post space preparation techniques with the DNS. This examination assesses many characteristics, such as canal deviation (coronal and apical deviation and angular deflection), volumetric loss in canal space, and operation time. The null hypothesis suggests no significant difference exists between DNS and conventional techniques in post space preparation during post endodontic management.

## Materials and methods

The study received approval from the local institutional scientific and ethical committee review board at SRM Hospital and Research Centre, Chennai, with the ethical clearance number SRMIEC-ST0723-1373. The sample size for this study was calculated considering a priori power analysis in G*Power software, version 3.1.9.2 (Heinrich Heine University, Düsseldorf, Germany). An alpha value of 0.05 and a statistical power of 95% were estimated with a total sample size of 60 (n). To avoid any unanticipated deviations from statistical assumptions, equal samples will be distributed in each of the two groups. 3D-DNS (group I; n = 30) and conventional techniques (group II; n = 30). The accuracy and efficiency between the two groups were evaluated by comparing the procedural time, global coronal and apical deviation, angular deflection, total volumetric loss of the instrumented root canal, and volumetric loss of the instrumented root canal 4 mm above the apex.

The tooth samples included in the present study were extracted due to the presence of periodontal disease. Before extraction, the patient consented to using the extracted tooth samples for the study. The inclusion criteria for selection of the experimental tooth were as follows: (1) intact teeth without any cavities or abnormalities, (2) teeth with a single root and straight canals, (3) radiographic verification of the presence of a single canal, and (4) fully matured roots. The research eliminated teeth that had previously had root canal therapy, significant tooth decay, dental restorations, dental crowns, fissures, or fractures, and teeth with noncarious lesions or immature apices. The samples used in this investigation were standardized by reducing 50% of the crown structure and ensuring that the root length was at least 13 mm. All teeth underwent sterilization using a steam autoclave and were subsequently stored in distilled water to maintain hydration.

Root canal preparation

Following access cavity preparation, a #15K file size (Dentsply Sirona, Ballaigues, Switzerland) was used to establish the working length. The working length was set to be 1 mm shorter than the apex of the root, as evaluated by radiographic examination. The canal was prepared biomechanically using a crown-down approach, using ProTaper Gold Rotary Files (Dentsply Sirona). The root canals were prepared up to the size of F3 ProTaper files (Dentsply Sirona) and flushed with 5 mL of 2.5% sodium hypochlorite after each file was used. Afterward, a volume of 5 mL of 17% ethylenediaminetetraacetic acid (EDTA) was used for irrigation. The canal was immersed in EDTA for one minute, and this process was repeated thrice within three minutes. Following this, the root canal was flushed with 5 mL of 2.5% sodium hypochlorite, and saline solution was used for final irrigation. After drying the canals with paper points (Dentsply Sirona), obturation was performed with F3 gutta-percha (Dentsply Sirona) and zinc oxide eugenol sealer. The tooth samples were then stored in an environment of 100% humidity at a temperature of 98.6°F (~37°C) for one week to facilitate the sealer set properly [7].

Preoperative CBCT and micro-CT scan and maxillary model

The 3D-printed maxillary cast was used to position the tooth sample. Six to seven experimental teeth were mounted within the maxillary cast, resulting in a total of nine models utilized. A preoperative single-arch CBCT scan (CS 9300; Carestream, Atlanta, GA) was conducted at a voxel size resolution of 0.120 mm³, and a preoperative micro-CT scan was taken for both groups. Following the preoperative CBCT and micro-CT scan, the teeth were allocated and categorized into two groups: DNS (group I; n = 30) and conventional groups (group II; n = 30).

DNS group

All procedures for both the DNS and conventional groups were conducted by a single operator, and the operator underwent training and calibration using DNS. The dynamic navigation process comprises three primary components. Initially, the CBCT information of the 3D-printed cast was imported into the DNS software in order to strategize the route for extracting the gutta-percha. Furthermore, registration was conducted to align the jaw of the cast model with the CBCT. This process included using a tracer instrument to precisely calibrate and designate specific points of reference on the teeth, facilitating registration without needing a stent. Before beginning the operation, the contra-angle handpiece, followed by the Peeso reamer, was calibrated in the third stage. Upon completion of registration, the system automatically transitioned to an accuracy verification process. The contra-angle handpiece and Peeso reamers were calibrated, and the system provided 3D views of the tooth, allowing monitoring of the location of the Peeso reamer tip. The post space preparation incorporated the utilization of Peeso reamers sequentially from number 1 to number 3. Each size of the Peeso reamer was calibrated using the DNS calibrator before proceeding with the next size.

**Figure 1:**
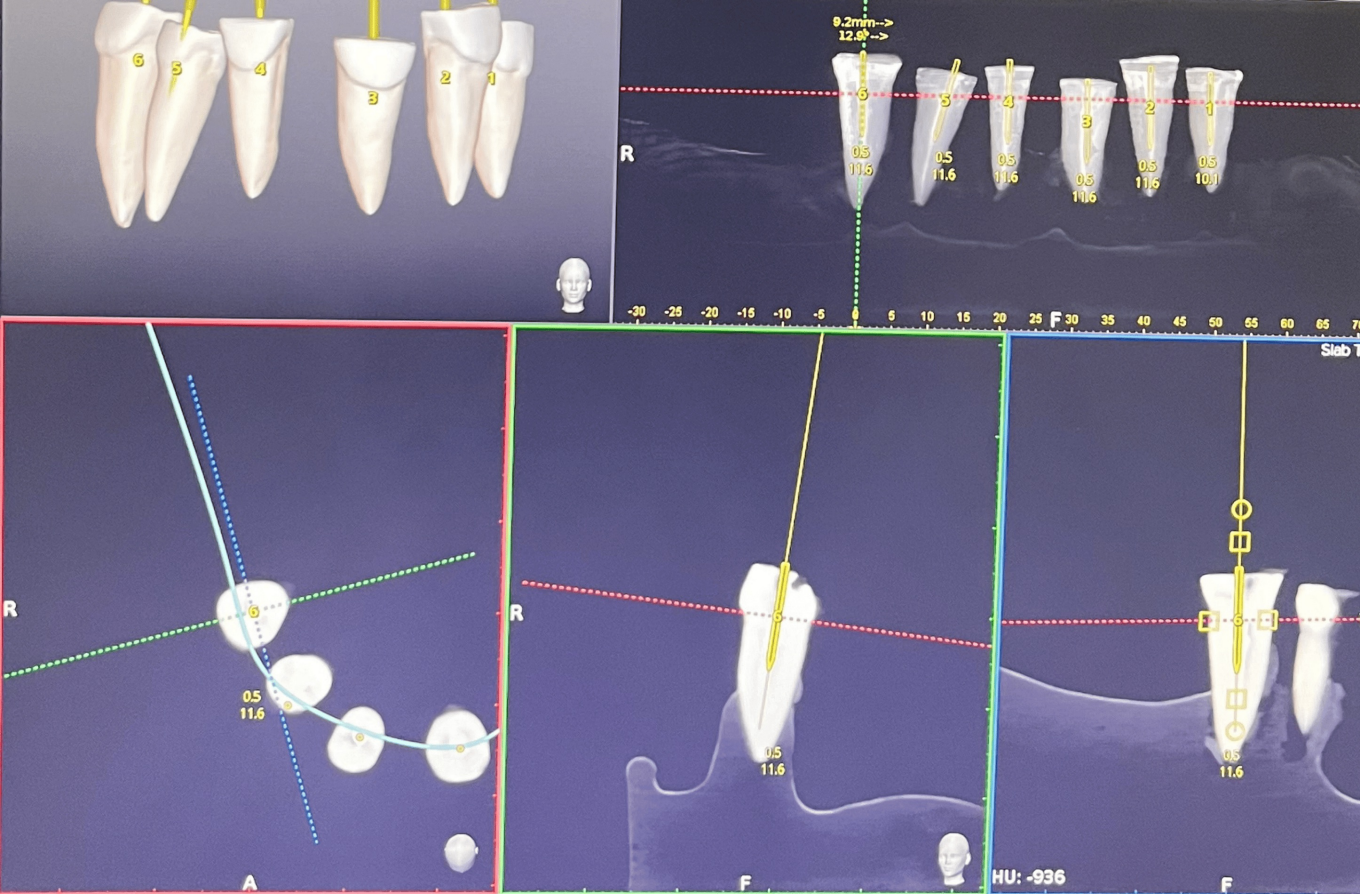
Planning for post space preparation under DNS DNS: dynamic navigation system

The entry point for drilling, trajectory, depth, and angle required for post space preparation was planned using DNS. To ensure standardization across both groups, Peeso reamers were utilized, with new reamers employed for individual teeth. Following trajectory planning and system calibration, under the complete guidance of the DNS using a contra-angle handpiece with the Peeso reamer connected to a micromotor, drilling through the gutta-percha was carried out. The drilling process was terminated upon reaching the length of the predetermined path, which is 4 mm short of the radiographic apex indicated on display in the system. The Navident software provided real-time feedback on the angulation and the correct position of the Peeso reamers during the drilling process. Figure 1 shows planning for post space preparation done under dynamic navigation in Navident software.

Conventional group

Post space preparation for the conventional technique was accomplished by measuring the length of the post space, which was established to be 4 mm short of the apex for each individual tooth based on the preoperative CBCT. Post space preparation was done using Peeso reamers from sizes 1 to 3, respectively, without the guidance of DNS. The time taken for post space preparation was evaluated using a timer. Postoperative CBCT and micro-CT were used for both groups. The canal deviation (coronal and apical deviation and angular deflection) was assessed by CBCT analysis, and volumetric loss of the instrumented root canal was estimated by micro-CT analysis.

Quantification of efficiency and accuracy

By overlaying pre- and postoperative CBCT scans, accuracy was assessed by using Evalunav software (ClaroNav Technology). Both groups were evaluated based on the complete removal of gutta-percha, overall deviation from the canal walls, and angular deflection from the preplanned trajectory for drilling in the preoperative CBCT scan to the achieved trajectory in the postoperative scan. The divergence of the canal's actual path from its planned position was measured using the universal deviation. The angular deflection determines the maximum angle between the central coordinates of the projected and actual drilling routes. The technician measured the deviations in X, Y, and Z dimensions without knowing the treatment specifics throughout the post space preparation and treatment planning stages. Pre- and postoperative micro-CT scans were used to analyze the total volumetric loss of instrumented canal space and volumetric loss of instrumented canal space 4 mm from the apex. The duration of the procedure, which was minutes, was used to evaluate efficiency. A blinded technician recorded the duration of post space preparation using a chronometer. The procedure's duration was measured for the DNS group from the commencement of post space preparation to the completion of the predetermined direction. In contrast, the duration utilized for the conventional group was recorded from the commencement of post space preparation until the Peeso reamer reached the predetermined length, which was indicated by inserting a stopper into the Peeso reamer.

Statistical analysis

Data regarding time (minutes), canal deviation (global coronal deviation in millimeters, global apical deviation in millimeters, and angular deflection in degrees), total volumetric loss of instrumented root canal, and volumetric loss of instrumented root canal above 4 mm from the apex (millimeters and in percentage) while using the dynamic navigation and conventional techniques were entered into Microsoft Excel (Microsoft Corporation, Redmond, WA) and analyzed using IBM SPSS Statistics for Windows, version 20 (IBM Corp., Armonk, NY). Data were investigated for normality using the Kolmogorov-Smirnov test. A normal distribution concerning all parameters except the total volumetric loss of the instrumented root canal and the volumetric loss of the instrumented root canal above 4 mm from the apex (in percentage) was observed. Descriptive statistics were derived from the mean, standard deviation, and confidence interval. Intergroup comparisons were analyzed using the independent t-test and Mann-Whitney U test, depending on the normality of the data. The level of statistical significance was determined at p < 0.05.

## Results

The present study examines three parameters: the duration of the procedure, the measurement of canal deviation using coronal and apical deviations, and angular deflection through CBCT analysis. Additionally, the study evaluates the total volumetric loss of the instrumented root canal and the volumetric loss of the instrumented root canal above 4mmm from the apex using micro-CT analysis.

Duration of the procedure

Table 1 illustrates the intergroup comparison of time taken for post space preparation between the dynamic navigation and conventional methods. The DNS group shows significantly less time taken than the traditional group. The average time taken for post space preparation in the dynamic group was (6.67 + 1.18 minutes) which was significantly less than the conventional group (17.23 + 2.16 minutes) (p < 0.05).

**Table 1:** Intergroup comparison of time (minutes) between the 3D dynamic navigation and conventional techniques ^a^Statistically significant (p < 0.05; independent t-test) df: degrees of freedom; 3D: three-dimensional

Parameter	Groups	N	Mean + standard deviation	Mean difference	95% confidence interval		df	Independent t-test value (p value)
					Lower	Upper		
Time (minutes)	3D dynamic navigation	30	6.67 + 1.18	-10.567	-11.467	-9.666	58	-23.491 (0.000)^a^
	Conventional technique	30	17.23 + 2.16					

CBCT analysis

Table 2 shows the CBCT results of the intergroup comparison of global coronal and apical deviations and angular deflection between 3D dynamic navigation and conventional techniques by superimposing preoperative and postoperative images using the DNS Evalunav software. The DNS group showed less canal deviation (global coronal and apical deviations and angular deflection) compared to the conventional group (p < 0.05).

**Table 2:** Intergroup comparison of mean ± standard deviation values for the global coronal and apical deviations and angular deflection between 3D dynamic navigation and conventional technique ^a^Statistically significant (p < 0.05; independent t-test). The global coronal and apical deviation and the angular deflection were used to determine the canal deviation 3D: three-dimensional

Measurement	3D dynamic navigation	Conventional technique	p value
Global coronal deviation (mm)	0.54 ± 0.13	0.90 ± 0.26	0.000^a^
Global apical deviation (mm)	0.57 ± 0.02	1.05 ± 0.29	0.000^a^
Angular deflection (degree)	0.85 ± 0.04	2.74 ± 0.75	0.000^a^

Micro-CT analysis

**Figure 2:**
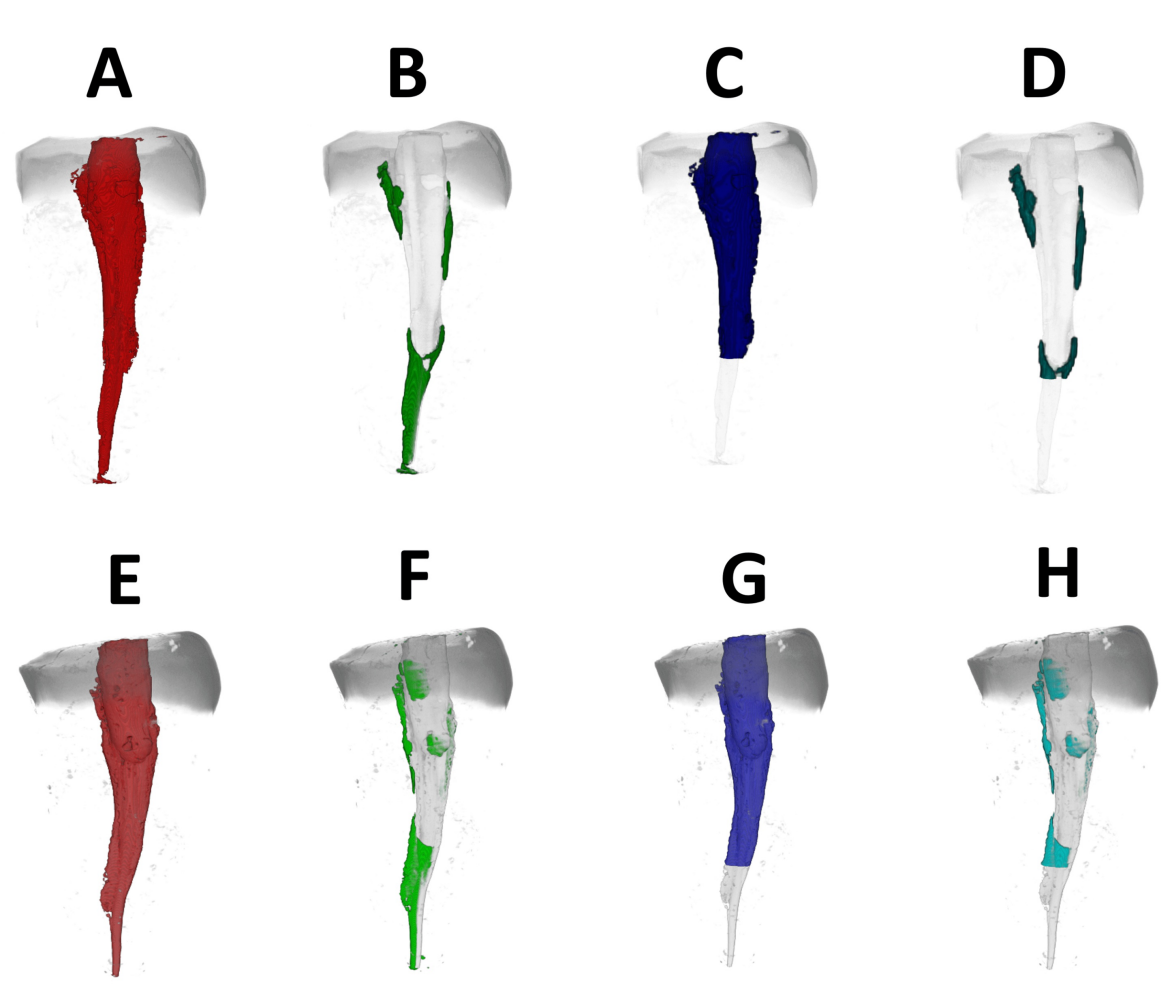
Pre- and postoperative micro-CT of groups I and II. (A) Preoperative micro-CT of the total volume of the root canal of group I. (B) Postoperative micro-CT of the total volume of the instrumented root canal of group I. (C) Preoperative micro-CT of the volume of the root canal above 4 mm from the apex of group I. (D) Postoperative micro-CT of the volume of the instrumented root canal above 4 mm from the apex of group I. (E) Preoperative micro-CT of the total volume of the root canal of group II. (F) Postoperative micro-CT of the total volume of the instrumented root canal of group II. (G) Preoperative micro-CT of the volume of the root canal above 4 mm from the apex of group II. (H) Postoperative micro-CT of the volume of the instrumented root canal above 4 mm from the apex of group II Group I: DNS Group II: conventional technique Micro-CT: micro-computed tomography

In this study, micro-CT analysis was done as it offers much superior resolution compared to CBCT. The enhanced resolution enables precise visualization of dental structures, such as root canals and their complicated architecture. Micro-CT provides a better evaluation of the volumetric loss of instrumented root canals than CBCT. Hence, micro-CT evaluation was used for this parameter of volumetric loss of the instrumented root canal. Figures 2A-2D show micro-CT images of group I, and Figures 2E-2H show micro-CT images of group II. Table 3 illustrates micro-CT results of the total volumetric loss of the instrumented root canal and volumetric loss of the instrumented root canal above 4 mm from the apex. The total volumetric loss of the instrumented root canal and the volumetric loss of the instrumented root canal above 4 mm from the apex are greater in the conventional group than in the DNS group (p < 0.05).

**Table 3:** Micro-CT results of volumetric loss of the instrumented root canal between the dynamic navigation and conventional groups ^a^Independent t-test ^b^Mann-Whitney U test ^c^Statistically significant (p < 0.05) The volumetric or substance loss is determined by subtracting the postoperative volume from the preoperative volume of the canal. The total volumetric loss of the canal and the volumetric loss of the canal above 4 mm from the apex were estimated. Values are determined using percentages as well. The results indicate a greater volumetric loss between groups II and I 3D: three-dimensional; micro-CT: micro-computed tomography

Measurement (preoperative and postoperative instrumentation)	3D dynamic navigation	Conventional technique	p value
Total volumetric loss of the instrumented canal space (mm^3^)^a^	15.95 ± 1.17	16.96 ± 1.12	0.001^c^
Total volumetric loss of the instrumented canal space (%)^b^	80.55 ± 1.87	87.04 ± 2.73	0.000^c^
Volumetric loss of the instrumented canal space 4 mm from apex (mm^3^)^a^	15.73 ± 1.22	17.08 ± 1.11	0.000^c^
Volumetric loss of the instrumented canal space 4 mm from the apex (%)​​​​​​​^b^	79.46 ± 1.90	87.63 ± 2.65	0.000^c^

## Discussion

Although previous studies have explored the concept of dynamic navigation, our study stands apart in that it is the first to include Peeso reamers directly in a DNS and the first to assess the accuracy and effectiveness of the DNS approach for post space preparation in post endodontic treatment.

The study's findings indicate that DNS is a more precise post space preparation method than traditional techniques. Additionally, DNS requires less time for operation than conventional techniques. The results of this study offer a comprehensive understanding of the effectiveness of a guided DNS in facilitating post space preparation on anterior teeth. This novel method is notably advantageous in real-time guided endodontics, as it considerably reduces the canal deviation (coronal and apical deviations and angular deflection) during gutta-percha removal compared to conventional procedures and considerably reduces the volumetric loss of tooth than the conventional technique. A noteworthy observation from the study is the considerable difference in the duration required for post space preparation between the DNS and conventional methods, with a mean difference of -10.567 minutes. Nevertheless, it is crucial to mention that the study did not include the time required for preparing guided access since this element has been examined in previous research investigations.

The accuracy of the DNS is assessed by measuring the global coronal and apical deviations and angular deflection by superimposing the preplanned drilling trajectory in the preoperative CBCT with the postoperative CBCT using the Evalunav software. The measured deviations are found to be 0.54 mm, 0.57 mm, and 0.85°, respectively. Meanwhile, for the conventional group, it is found to be 0.90 mm, 1.05 mm, and 2.74°. The angular deflection seen in this study is 0.85°, which is lower when compared to previous studies that investigated the accuracy of dynamic navigation in intraosseous anesthesia [8] and calcified canals [9], which reported angular deflections of 1.36° and 1.69°, respectively.

Real-time guided dynamic navigation is considered more precise and efficient than the conventional method in identifying calcified canals. Between the computer-aided dynamic and static navigation techniques, there are no statistically significant differences in success rate for the location of root canals [10]. In recent times, Dianat et al. [11] evaluated the precision of dynamic navigation in root-end resection. They found that the DNS group had a 2.54° angular deflection, whereas the Freehand group had a deflection of 12.38°. The data collected in this study and earlier research [6,8,9,11-14] suggest that the DNS is highly accurate in endodontic procedures. It should be noted that the effectiveness of the DNS procedure relies on the expertise of the operator [13,15]. The DNS has a resolution of 0.5° and 0.25 mm for tracking the movement of the bur and handpiece [15]. Hand trembling, even at modest levels, can have an impact on the precision of the procedure.

In future research, Jain et al. warranted using advanced segmentation approaches and 3D image processing (micro-computed tomographic imaging) to detect better 3D accuracy [13]. Therefore, a micro-CT analysis was also included in the present study to evaluate the substance loss or total volumetric loss of the instrumented root canal and volumetric loss of the instrumented root canal above 4 mm from the apex. The results are found to be 15.95 ± 1.17 mm^3^ (80.55% ± 1.87%) and 15.73 ± 1.22 mm^3^ (79.46% ± 1.90%), respectively, for the DNS group and 16.96 ± 1.12 mm^3^ (87.04% ± 2.73%) and 17.08 ± 1.11 mm^3^ (87.63% ± 2.65%), respectively, for the conventional group. This depicts that there is less volumetric loss of the instrumented root canal in the DNS group compared to the conventional group. The current investigation supports the findings of a systematic review and meta-analysis carried out by Mekhdieva et al., which indicate that the dynamic navigation approach appears to yield better results for both microsurgical and nonsurgical endodontic procedures, such as reduced substance loss, shorter operating times, and a lower risk of iatrogenic complications. Additionally, the protocol improves the precision of treatment [16].

This research emphasizes the precision and efficacy of dynamic navigation in facilitating post space preparation on anterior teeth. Dynamic navigation provides a conservative approach with substantial clinical benefits by minimizing canal deviation and preserving the integrity of tooth structure by reducing the volumetric loss of instrumented root canals. Moreover, the reliability of DNS is further improved by its independence from the operator's level of expertise, even for inexperienced dentists. The research conducted by Tang and Jiang demonstrates that the operator's degree of expertise has no impact on dynamic navigation, therefore supporting this finding. This feature emphasizes the dependability of dynamic navigation, making it an appealing alternative for endodontic procedures, regardless of the operator's level of expertise [4]. These discoveries provide important insights into the field of endodontics and emphasize the potential of dynamic navigation as a standard practice in future endodontic treatments.

In general, the DNS workflow is straightforward, uncomplicated, and readily integrated with existing procedures. The dynamic navigation technique initially depends on the preplanning accuracy and quality of the CBCT scan, as well as the stability of the fiducial for the scan. Collectively, the DNS studies that have been included indicate that dynamic navigation is a prospective tool for various procedures in endodontics [17]. The entire procedure being controlled in real-time makes DNS safer, less intrusive, and more precise, as it eliminates potential errors associated with the creation and positioning of the guide [18].

By registering the dentition and reconstructing the CBCT findings, the dental professional may precisely track the placement of the drills on the CBCT reconstruction using specialized software and tracking techniques. This allows for monitoring the rotary motion of the treatment zone and devices. Consequently, any variations in the drill may be monitored and resolved. Moreover, it enables us to modify the virtual deliberation during the process [19].

Dynamic navigation may further enhance patient education by ensuring comfort and transparency throughout the treatment, reducing patient anxiety altogether. Nevertheless, it is crucial to acknowledge that dynamic navigation should not be considered a substitute for meticulous surgical planning and precise execution performed by a proficient and seasoned dental practitioner [20].

Study limitations

The maxillary central incisor is selected because the DNS has some limitations in requiring straight access to the canal until the apex. Further studies need to be done with more calibrated DNS for multiple rooted and abbreviated canals to check its efficiency in anatomically variated teeth. The study has some limitations despite the precision of DNS, including the complication of observing the system display during the treatment compared to direct vision. However, looking at the system display might be ergonomically beneficial. Another limitation is that a large tracker attachment for the handpiece causes discomfort during regular use, especially for treatment in the posterior region. Certain firms have devised methods such as dynamic reference frames by laser printing into the body of the handpiece or making adjustments to the design of an attachment to enhance grip. The machine's relatively high cost can hinder the extensive adoption of this technology. Nevertheless, the expense would be adequately justified if it shows to effectively save substantial procedure time and minimize accidental errors in the clinical context. In the near future, the restrictions of adopting advanced technologies like augmented reality and design adjustments can be solved [12].

Another limitation of this study is that DNS has a long learning curve. Moreover, a clinical trial by Stefanelli et al. demonstrated the significance of the long learning curve associated with this technology. It revealed a noteworthy enhancement in the average coronal and apex deviations and angular deflection after the completion of the initial and last 50 implant surgeries. Therefore, the operator's accuracy outcomes seem to improve as their expertise level with dynamic navigation increases [21].

## Conclusions

Based on the constraints of this laboratory research, it is possible to infer that the dynamic navigation approach under investigation results in a more precise post space preparation, substantially less canal deviation, reduced volumetric loss, and a shorter procedure time than the conventional method. Dynamic navigation is regarded as a viable technique because of its highly predictable outcome, substantially reduced operation time, and reduced risk of iatrogenic injury. The advent of guided navigation in endodontics has enabled dentist to eliminate the need for superfluous tissue excision during location of the root canal, decrease the likelihood of mishaps, enhance the treatment outcomes, and reduce the duration of the procedure, even for the inexperienced dentists.
